# Safety and feasibility of faecal microbiota transplant for major depressive disorder: study protocol for a pilot randomised controlled trial

**DOI:** 10.1186/s40814-023-01235-z

**Published:** 2023-01-09

**Authors:** Jessica E. Green, Amelia J. McGuinness, Michael Berk, David Castle, Eugene Athan, Christopher Hair, Philip Strandwitz, Amy Loughman, Andrew A. Nierenberg, John F. Cryan, Mohammadreza Mohebbi, Felice Jacka

**Affiliations:** 1 Deakin University, Food & Mood Centre, IMPACT-the Institute for Mental and Physical Health and Clinical Translation, School of Medicine, Barwon Health, Geelong, Australia; 2 Monash Alfred Psychiatry Research Centre (MAPrc), Central Clinical School, Faculty of Medicine Nursing and Health Sciences, Monash University, Melbourne, Australia; 3 Department of Psychiatry, Peninsula Health, Frankston, Australia; 4 Department of Psychiatry, University of Melbourne, Parkville, Australia; 5 Orygen Youth Health Research Centre and the Centre of Youth Mental Health, Melbourne, Australia; 6 The Florey Institute for Neuroscience and Mental Health, Parkville, Australia; 7 Barwon Health, Geelong, Australia; 8 Centre for Addiction and Mental Health and Department of Psychiatry, University of Toronto, Toronto, Canada; 9 School of Medicine, Deakin University, Geelong, Australia; 10 Holobiome, Inc., Boston, MA USA; 11 Dauten Family Center for Bipolar Treatment Innovation, Department of Psychiatry, Massachusetts General Hospital, Boston, MA USA; 12 Harvard Medical School, Boston, MA USA; 13 Department of Anatomy and Neuroscience, University College Cork and APC Microbiome, Cork, Ireland; 14 Centre for Adolescent Health, Murdoch Children’s Research Institute, Royal Children’s Hospital, Parkville, Australia; 15 Black Dog Institute, Melbourne, Australia; 16 James Cook University, Townsville, Australia

**Keywords:** Faecal microbiota transplantation, Microbiome, RCT, Psychiatry, Mental disorder, Neuroscience, Depression, Major depressive disorder, Mood disorders, Mental health

## Abstract

**Background:**

Mental disorders, including major depressive disorder (MDD), are a leading cause of non-fatal burden of disease globally. Current conventional treatments for depression have significant limitations, and there have been few new treatments in decades. The microbiota-gut-brain-axis is now recognised as playing a role in mental and brain health, and promising preclinical and clinical data suggest Faecal Microbiota Transplants (FMT) may be efficacious for treating a range of mental illnesses. However, there are no existing published studies in humans evaluating the efficacy of FMT for MDD.

**Methods and design:**

This protocol describes an 8-week, triple-blind, 2:1 parallel group, randomised controlled pilot trial (*n* = 15), of enema-delivered FMT treatment (*n* = 10) compared with a placebo enema (*n* = 5) in adults with moderate-to-severe MDD. There will be a further 26-week follow-up to monitor longer-term safety. Participants will receive four FMT or placebo enemas over four consecutive days. The primary aims of the study are to evaluate feasibility and safety of FMT as an adjunctive treatment for MDD in adults. Changes in gut microbiota will be assessed as a secondary outcome. Other data will be collected, including changes in depression and anxiety symptoms, and safety parameters.

**Discussion:**

Modification of the microbiota-gut-brain axis via FMT is a promising potential treatment for MDD, but there are no published rigorous clinical trials evaluating its use. If this study finds that our FMT strategy is safe and feasible, a larger fully powered RCT is planned. Further high-quality research in this field is urgently needed to address unmet need.

**Trial registration:**

Australian and New Zealand Clinical Trials Registry: ACTRN12621000932864

## Background

Mental disorders, including major depressive disorder (MDD), are a leading cause of non-fatal burden of disease globally [1]. Current evidence-based treatments for MDD (i.e. antidepressants and psychotherapy) have limitations including limited efficacy, side effects, and cost, as well as access issues in the case of psychotherapy. Moreover, the economic pressure of mental disorders supersedes that of cancer, diabetes, and cardiovascular diseases [2]. Thus, the development of affordable and novel treatments for MDD that can augment existing strategies would be of significant benefit to patients and their families, to the healthcare system, and to the broader economy.

### Microbiota-gut-brain-axis

There is a well-established association between mental health and the gut microbiota that co-exist symbiotically in our gastrointestinal tract, referred to as the ‘microbiota-gut-brain axis’ [3, 4]. There are data linking various biological variables to the aetiology and maintenance of MDD; these have been extensively reviewed elsewhere, and include inflammatory mediators (e.g. elevations in c-reactive protein and inflammatory cytokines), metabolic factors (e.g. insulin resistance, metabolic syndrome), oxidative stress (e.g. reactive oxygen species and mitochondrial dysfunction), the hypothalamic-pituitary-adrenal (HPA)-axis (e.g. perturbations in cortisol), neurotransmitters (e.g. gamma-aminobutyric acid and serotonin precursors), neuropeptides (e.g. brain-derived neurotrophic factor (BDNF)), and other systems [3, 5]. Each of these systems are potentially influenced by the gut microbiota, and emerging evidence linking gut microbiota to brain and behaviour suggests that the gut may be a key modifiable target for mental and brain health [3].

### Evidence for the potential role of gut microbiota in treating MDD

Differences in gut microbiota composition have been observed in depressed compared to non-depressed humans across several studies [4, 6–10]. Commonalities across studies include increased lactic acid producing bacteria such as *Lactobacillus*, *Streptococcus*, and *Enterococcus*, and reductions in bacteria with the capacity to produce the short-chain fatty acid butyrate, such as *Faecalibacterium* and *Coprococcus* [4, 11]. Additionally, there appears to be evidence of correlations between specific gut bacteria and depression symptomatology [4, 12]; however, few studies have explored this link, and the associations between MDD symptomatology, gut microbiota composition, and their functional potential are poorly understood.

Further to this, there is evidence supporting the potential efficacy of probiotics for depression and anxiety [13]. Meta-analyses of studies investigating probiotic supplementation as an adjunctive treatment for depression suggest that this treatment strategy may be effective for the reduction of depressive symptoms, particularly in those with clinical depression [14, 15], or alongside antidepressant therapy [16]. However, these treatments are limited to a single bacterial strain or, at best, a small number of strains. Conversely, faecal microbiota transplant (FMT), which encompasses a complete human gut microbiome containing thousands of potentially symbiotic strains [17], may have the potential to more effectively alter the composition of the gut microbiota, thus the influence of FMT on mental health symptoms warrants further exploration.

### preclinical evidence supporting a trial of FMT for MDD

Experimental studies in rodents suggest that alterations of microbial composition of the gut via FMT can induce changes in behaviour [18, 19] and influence rodent models of psychiatric disorders [20]. A seminal study by Kelly et al. [7] provided the first evidence that the transfer of faecal matter from individuals with MDD into germ-free rodents could induce depression-like behaviours in the rodents, as well as other physiological features associated with depression such as changes in tryptophan metabolites. Remarkably, four further studies have now shown that the transfer of faeces from depressed humans into microbiota-depleted mice induces depression-like behaviours in the mice [21–24], and similar outcomes have also been observed in rodent models of schizophrenia [25]. Together, these preclinical studies provide foundational evidence of the potential utility of FMT for depression.

### Evidence for FMT in neuropsychiatric disorders in humans

In humans, FMT is an established treatment for *Clostridioides difficile* infection (CDI) [26, 27], with a cure rate of 80–90%. It has also shown potential in a variety of other conditions [28–30], including gastrointestinal [31–35], autoimmune [36, 37], antibiotic-resistant organisms, hepatic disorders, metabolic [38, 39], and neuropsychiatric conditions [40, 41] including autism spectrum disorder [41]. Whilst to date no large scale studies in humans have investigated the use of FMT for psychiatric disorders, case studies suggest promising outcomes with respect to the treatment of depressive symptoms [42, 43] and bipolar disorder [44, 45]. Studies of FMT in people with irritable bowel syndrome [IBS]—which is commonly comorbid with psychiatric symptoms—have also shown improvements in mental health symptomatology [28, 40, 46, 47]. Case studies have also reported a reduced need for antidepressant medications in IBS patients post-FMT [48].

### safety of FMT

The safety of FMT has been established across multiple patient populations [29], including immunocompromised [49] and paediatric populations [50], and over the long term [51]. Other studies have demonstrated FMT to be safe even in healthy recipients, not merely those with CDI and other health disorders [52]. Indeed, adverse reactions are rare and appear to be more frequently associated with delivery method [53–55] or the underlying disorder and/or symptoms for which FMT is being used [28], rather than the FMT itself. Indeed, our recent systematic review and meta-analysis of FMT studies for conditions other than CDI found more serious adverse events reported in the placebo group than in the group receiving the FMT intervention [28]. Of the adverse events observed in participants allocated to FMT, all but one such event was deemed unlikely to be related to the FMT. Moreover, rates of mild to moderate adverse events were similar between participants receiving FMT and those who received placebo.

Whilst most FMT studies report only mild and self-limiting adverse events [56], in 2019 a warning regarding the risks of FMT was released by the US Food and Drug Administration (FDA). This warning was in response to a death that occurred in an immunocompromised patient who received unscreened donor faeces containing antibiotic-resistant organisms, specifically, extended spectrum beta lactamase-producing *Escherichia coli*. This serious incident highlights the importance of adhering to rigorous donor screening protocols including screening thoroughly for antibiotic-resistant organisms. In Australia, the practice of FMT is now regulated by the Therapeutic Goods Administration (TGA), which includes strict, detailed, and comprehensive standards for the manufacture and supply of FMT. The FMT used in this study (the “Moving Moods Pilot Study”) utilises a product that meets the TGA standards.

## Methods

### Objectives

The primary objectives for this study are to evaluate the feasibility and safety of FMT as an adjunctive treatment for MDD in adults. The secondary objective is to evaluate the impact of enema-delivered FMT on gut microbiota compared with a placebo enema in adults with MDD. As this study is not powered to evaluate efficacy, exploratory objectives include any changes observed in depression and anxiety symptoms, sleep, quality of life, level of function, gut symptomatology, blood biomarkers of neurogenesis, gut permeability, inflammation, and cardiometabolic parameters following delivery of FMT via enema compared with a placebo enema in adults with depression.

### Study design

This study is an 8-week, triple-blind, 2:1 parallel group, randomised controlled pilot trial (*n* = 15), of enema-delivered FMT (*n* = 10) compared with a placebo enema ( *n*= 5) in adults with moderate-to-severe MDD. There will be a further 26-week follow-up for safety data.

### Study intervention

Participants will receive a total of four doses of an FMT via enema (henceforward referred to as ‘active’), or four doses of placebo enema, as an adjunct to treatment as usual. The active FMT enema will comprise of syringes supplied by BiomeBank containing a total volume of 50 mL including 12.5 mg donor faeces: normal saline (0.9%) and 10% glycerol. The FMT was prepared in accordance with the Australian TGA standards which are described in detail on the TGA website (TGO-105: Standards for FMT) [57]. Briefly, anonymous, fit, and healthy volunteers aged 18–60 years undergo a rigorous screening process prior to donation, including a thorough medical interview and examination by a physician, and the collection of donor biological samples (stool, blood, and nasal swab) to ensure the absence of viral, bacterial, and parasitic pathogens. For a full overview of the donor screening process, see reference [58] (TGO-105: Standards for FMT) [57]. The placebo will consist of a visually identical placebo product containing a total volume of 50 mL including normal saline; 10% glycerol, and brown dye.

### Procedure

**Fig. 1:**
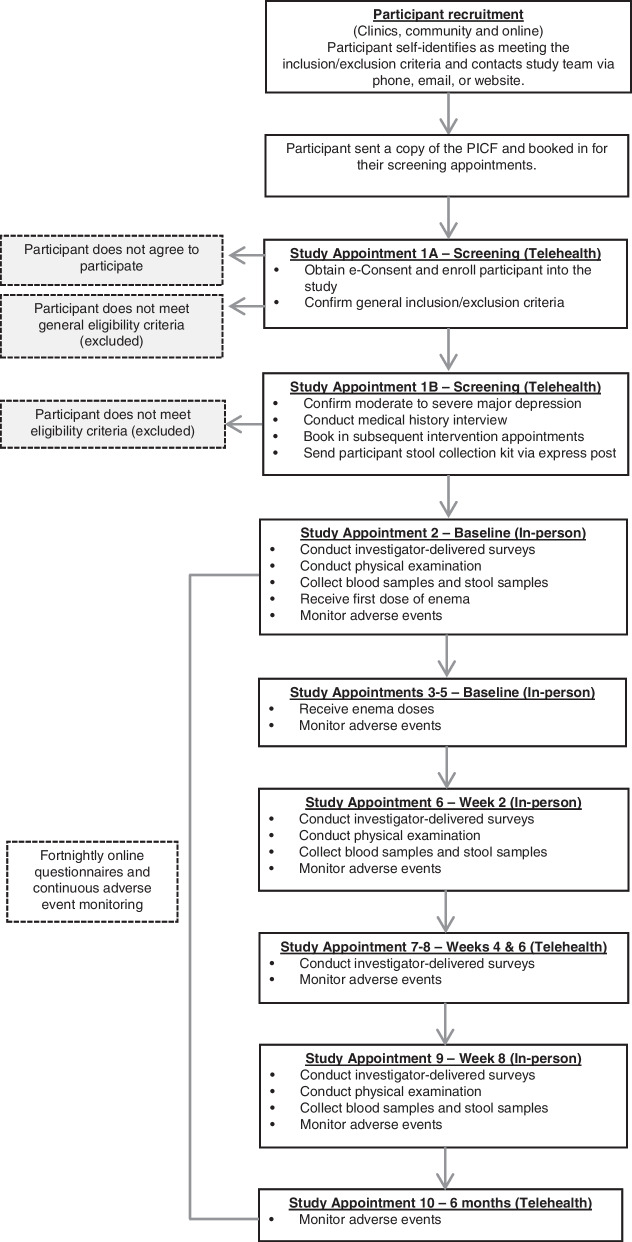
Appointment schedule

Participants will receive either four active or four placebo FMT delivered via an enema will be administered over four consecutive days to each participant by a research nurse. Assessments will be conducted at baseline, and then every 2-weeks until the primary end point (8 weeks). A further follow-up assessment will then occur at week 26 to capture long-term safety data. An outline of the study schedule is provided in Table 1 and the appointment schedule is summarised in Fig. 1. Participants, investigators, assessors, and study statisticians will be blinded to group allocation. This protocol is reported as per the Standard Protocol Items: Recommended for Intervention Trials (SPIRIT) guidelines [58], and the CONSORT extension for pilot and feasibility trials checklist [59]. All study procedures will be concordant with Good Clinical Practice (GCP) principles. The consent procedure is described below under the heading Consent and Enrolment.

**Table 1:** Schedule of assessments, instruments, and procedures to be implemented at each study appointment

Variable	Instrument	*Week (first row)* Appointment number (second row)										
		*− 2*		*0 (baseline)*				*2*	*4*	*6*	*8*	*26*
		1a	1b	2	3	4	5	6	7	8	9	10
Screening (via Telehealth)												
Enrolment	Informed consent	✓										
General inclusion criteria	Eligibility screener for entrance criteria	✓										
Alcohol and substance abuse	Alcohol Use Disorders Identification Test (AUDIT) Drug Abuse Screening Test (DAST-10)	✓										
Diagnosis of MDD	Structured Clinical Interview for the DSMI-V (SCID-V) mood disorders module		✓									
Depression severity and suicidality	Montgomery-Asberg Depression Rating Scale (MADRS)		✓									
Study appointments												
Group allocation	Randomisation spreadsheet			✓								
FMT	Enema-delivery of intervention or placebo			✓	✓	✓	✓					
Demographics	General questionnaire			✓								
Medical history	Medical history Standard psychiatric assessment Family history		✓ (optional)	✓								
Study appointments continued.												
Medication use	Current medications Medication history		✓	✓								
Personality disorders	Personality disorders (SAPAS)			✓								
Diet	Diet (Simple Dietary Questionnaire)			✓							✓	
Psychological symptoms	Depression, Anxiety and Stress symptomatology (DASS) Depression severity (MADRS) Suicidality (MADRS)			✓				✓	✓	✓	✓	
Gut symptoms	Gastrointestinal Symptom Rating Scale (GSRS)			✓				✓	✓	✓	✓	
Medical variables	Change in medications, vaccines, illness, etc			✓				✓	✓	✓	✓	
General health and functioning	Patient Global Impression of Change Sheehan Disability Scale Sleep (Pittsburgh Sleep Quality Index) Quality of Life			✓				✓	✓	✓	✓	
Study appointments continued.												
Changes of circumstance	Medication changes questionnaire			✓				✓	✓	✓	✓	
Cost analysis	Resource Utilisation Questionnaire			✓							✓	
Physical examination	Height (stadiometer) Weight (scales) Blood pressure (sphygmomanometer) Heart rate (sphygmomanometer)			✓				✓			✓	
Blood samples	Whole blood by research nurse/ACL			✓				✓			✓	
Stool samples	Microba stool collection kits			✓				✓			✓	
Feasibility data												
Tolerability	Adverse events			✓	✓	✓	✓	✓	✓	✓	✓	✓
Adequacy of blinding	Participant questionnaire							✓				
Adherence	Monitoring questionnaire completion and appointment attendance throughout the study period			✓	✓	✓	✓	✓	✓	✓	✓	
Acceptability	Study Participant Feedback Questionnaire Specific questions pertaining to study intervention			✓				✓			✓	
Researcher/trial feasibility data												
Recruitment	Monitoring of recruitment rates by study staff throughout trial period			✓	✓	✓	✓	✓	✓	✓	✓	
Retention	Monitoring of trial attrition by study staff throughout trial period			✓	✓	✓	✓	✓	✓	✓	✓	

The study schedule allows for flexibility for all or parts of each appointment to be conducted by telehealth if this is preferable to the participant or required for other reasons

### Study setting

The trial will be conducted at a dedicated clinical trials facility at University Hospital Geelong, Victoria. This space includes appropriate consultation spaces and on-site equipment for intervention delivery and the collection of biological samples. This study will consist of 10 study appointments, six of which are in-person and four via telehealth (see Table 1 and Fig. 1).

The collection of blood samples will be performed by a qualified research nurse or phlebotomist, where markers of cardiovascular and metabolic disease risk (e.g. lipids, HbA1c, and blood glucose) will be assayed. Additional blood samples will be collected and transported to the Geelong Centre for Emerging and Infectious Disease (GCEID) laboratories in Geelong for additional blood analyses and long-term storage.

Stool samples will be collected by participants at home using a Microba (Queensland, Australia) stool sampling kit. Participants will then return the completed kits to research personnel at their study appointments. These stool samples will be de-identified (marked with a participant code only) and sent to Microba for metagenomic analyses.

### Study population

This study aims to recruit 15 participants, aged 18 to 65 years of age, with a DSM-5 diagnosis of MDD (confirmed by a psychiatrist using the Structured Clinical Interview for DSM-5 (SCID-5) MDD module) [60] of moderate-to-severe range (defined as a Montgomery Asberg Depression Rating Scale (MADRS) score of greater than or equal to 20) [61]. Eligibility criteria are outlined in Table 2. The recruitment strategies are outlined in Table 3. Once participants have given consent and been screened and determined eligible for entry into the study, they will be randomised to either active FMT enema or placebo enema, which will be delivered on four consecutive days in the first week of the study. Participants will then be followed up fortnightly for 8 weeks, with a final appointment at week 26 to capture long-term safety data. The participant schedule and study outcomes are summarised in Table 1 and Fig. 1

**Table 2:** Eligibility criteria of participants enrolled in the Moving Moods Pilot Study

Inclusion criteria:	
1. Adults (age 18–65 years)	
2. MDD according to Structured Clinical Interview for DSM-5 (SCID-5) MDD module	
3. Moderate-to-severe score on MADRS (i.e. score of greater than or equal to 20)	
4. Stable treatment (i.e. pharmacological and psychological) for 1 month prior to commencing trial	
Exclusion criteria:	
1. Active suicidality (a MADRS suicide item score of 5 or 6)	
2. Use of probiotics, antibiotics, or any experimental drug in the 1 month prior to study entry	
3. Serious gastrointestinal conditions (including active inflammatory bowel disease, bowel cancer, or a history of major bowel surgery, but not including IBS, chronic diarrhoea, or constipation)	
4. Pregnancy or breastfeeding (pregnancy will be excluded using a urine pregnancy test at baseline)	
5. Major comorbid psychiatric disturbances including bipolar disorder, a primary psychotic illness, obsessive-compulsive disorder, anorexia nervosa, or bulimia nervosa	
6. Active substance-use disorder, defined as a score of 6 or greater on the brief Drug Abuse Screening Test (DAST-10), and/or a score of 16 or greater in the Alcohol Use Disorders Identification Test (AUDIT)	
7. Inability to read and understand the participant information and informed consent form	
8. Patients with a history of severe anaphylactic or anaphylactoid food allergy	
9. A condition that could jeopardize the safety or rights of the subject, make it unlikely for the subject to complete the study, or confound the results of the study

**Table 3:** Recruitment strategies

1. Flyers will be displayed in the waiting rooms of key recruitment sites	
2. Advertisements through social media	
3. Clinicians at key recruitment sites will advise of appropriate patients for the trial	
4. Trial Facts or HealthShare, which are services connecting patients and doctors, will identify appropriate participants for the trial. These will be utilised only if the above strategies are insufficient.	

### Follow-up appointments

Follow-up appointments will be conducted by a qualified member of the research team. The data collected at these assessments are summarised in Table 1.

### Outcomes

The study appointment schedule is detailed in Table 1. Primary, secondary, and exploratory outcomes are described below.

### Primary outcome measures

The primary outcomes for this study are (1) the feasibility and (2) safety of FMT as an adjunctive treatment for MDD in adults. The study outcomes, participant appointment schedule, and study timeline are outlined in Table 1 and Fig. 1.

Feasibility will be considered as a composite outcome, measured by the following:

- The ability to meet recruitment targets (measured by recruitment logs, comparing actual recruitment against projected targets)
- Participant retention and completion rates (measured by attrition rates and completeness of data)
- Adherence to intended protocol (measured by completion of intervention as planned, missed appointments, technical difficulties arising, and completeness of study data)
- Participant acceptability (measured using the TransCelerate Study Participant Feedback Questionnaire, plus the addition of questions designed specifically for this study)
- Robustness of study methodology, including effectiveness of blinding (measured qualitatively based on feedback from researchers throughout and at the conclusion of the study, and a participant and researcher survey to assess blinding)

Safety of FMT as an adjunctive treatment in adults will be measured by an assessment of adverse events. These data will be collected through direct observations by the study nurse during administration of the intervention, and via participant reporting during the intervention and each appointment thereafter (measured at weeks 0, 2, 4, 6, 8, and 26). Participants will also be encouraged to contact the research team at any time to report adverse events outside of study appointments.

### Secondary outcome measures

The secondary outcome measure of this study is changes in gut microbiota composition and functional potential, which will be measured via the following:

1. The degree of change in gut microbiota composition and functional potential in recipients, which will be assessed by observing changes in gut microbiota composition (alpha diversity, beta diversity, differential abundance of species) and functional potential at 2- and 8-weeks post-intervention compared with baseline.
2. The degree of microbial ‘engraftment’: degree to which the changes in gut microbiota composition and functional potential of recipients are concordant with the gut microbiota profiles of the donor stool post-active FMT and compared with placebo enema.
  - Donor vs recipient at baseline (0 weeks)
  - Donor vs recipient at 2 weeks (active FMT compared with placebo group)
  - Donor vs recipient at 8 weeks (active FMT compared with placebo group)

### Exploratory outcome measures

The following data will be collected as part of the primary feasibility outcome, described above. However, given the small sample size, it is expected that the study will be underpowered to measure significant changes in any of the following outcome measures. As such, these outcomes are considered “exploratory”. To avoid type I error inflation due to multiple comparisons, we aim to only record summary descriptions of these clinical outcomes. These exploratory outcomes are as follows:

1. Between-group differential change from baseline and weeks 2, 4, 6, and 8 in mental health symptoms as measured by the Montgomery-Asberg Depression Rating Scale (MADRS) and the Depression-Anxiety Stress Scale (DASS)
2. Between-group differential change from baseline and weeks 2, 4, 6, and 8 in quality of life as measured by the Assessment of Quality of Life-8 Dimension (AQoL-8D); this scale also allows the calculation of preference-based outcomes also known as utilities. Utility values will be used to calculate quality adjusted life years (QALYs), a common metric used in economic evaluations
3. Between-group differential change from baseline and weeks 2, 4, 6, and 8 in sleep as measured by the Pittsburgh Sleep Quality Index
4. Between-group differential change from baseline and weeks 2, 4, 6, and 8 in level of function as measured by the Sheehan Disability Scale
5. Between-group differential change from baseline and weeks 2, 4, 6, and 8 in gut symptomatology as measured by the Gastrointestinal Symptom Rating Scale (GSRS)
6. Between-group differential change from baseline and weeks 2 and 8 in in blood biomarkers of neurogenesis (e.g. brain-derived neurotrophic factor)
7. Between-group differential change from baseline and weeks 2 and 8 in cardiometabolic blood parameters (e.g. random lipid profile, random blood sugar levels and HbA1c)
8. Between-group differential change from baseline and weeks 2 and 8 in metabolic and cardiovascular risk factors assessed via physical exam, including heart rate, blood pressure, height, and weight
9. Cost effectiveness will be assessed from both health sector and societal perspectives. The cost of FMT via enema will be estimated and added to the cost of lost productivity and health care resources utilised by participants over the course of the trial using the Resource Utilization Questionnaire (measured at weeks 0 and 8)
10. Between-group differential change from baseline and weeks 2, 4, 6, and 8 in self rated overall improvement assessed using the Patient Global Impression of Change
11. Between-group differential change from baseline and weeks 2 and 8 in inflammation (e.g. macrophage inhibitory factor, interleukins 1b, 1ra, 6 and 10, soluble CD14, and high sensitivity C-reactive protein) will be measured in plasma and/or serum using immunoassays (e.g. solid phase sandwich ELISA assay)
12. Between-group differential change from baseline and weeks 2 and 8 in gut permeability (e.g. lipopolysaccharide binding protein and zonulin) will be measured in plasma and/or serum using immunoassays (e.g. solid phase sandwich ELISA assay)

### Feasibility targets

Table 4 outlines pre-specified targets identified by the research team as a minimum standard that would indicate that the study design was feasible. These targets will also assist the study team to identify aspects of the trial that may need to be amended to improve future, large-scale trials.

**Table 4:** Feasibility targets

Target area	Feasibility target
Recruitment	Successful enrolment of *n* = 15 participants across a 6-month active recruitment period
Retention	A minimum of *n* = 10 participants to complete study until the 8-week primary endpoint (i.e. a 33% attrition rate).
Adherence to protocol	Participants should: 1) Receive two of the total four enemas 2) Attend their baseline, week 2 and week 8 appointments 3) Provide baseline and week 2 stool samples
Safety	Nil severe and/or serious adverse events rated as likely due to study intervention in the active FMT group.
Adequacy of blinding	The best achievable outcome for adequacy blinding is participants and researchers correctly guessing allocation at a rate of 50% (the rate due to chance). Whilst we are not likely to be statistically powered to measure this outcome, we aim to reach an outcome approaching 50%.

### Sample size, power, and statistics

#### Feasibility analysis

As this is a feasibility study, it is not designed or powered to evaluate statistically significant changes in any of the parameters being assessed. Therefore, a qualitative synthesis of feasibility data will be reported.

#### Gut microbiota analyses

Longitudinal analysis of changes in gut microbiome composition will be performed within the RStudio [62] environment. Measures of gut microbiota alpha-diversity (e.g. Shannon Index, Simpson Index, Chao-1, ACE, evenness, Faith’s phylogenetic diversity) [63, 64] and beta-diversity (e.g. Bray-Curtis, Jaccard, Aitchison distance) [65] will be calculated for donors (baseline) and recipients (three timepoints: baseline, 2 weeks, 8 weeks post-intervention). Differences in alpha-diversity between donors and recipients (between groups) will be tested using the Mann-Whitney test and between each time point (within groups) will be compared using Kruskal-Wallis non-parametric test. An appropriate method will be used to correct for multiple comparisons. Permutational multivariate analysis of variance (PERMANOVA) and visualization with principal coordinate analysis (PCoA) will be used to estimate any differences in gut microbiota community structure (beta-diversity) between donors and recipients and in recipients over time. Differential abundance analyses will be conducted to compare the taxonomic composition of gut microbiota between donors and recipients and in recipients over time.

### Trial allocation, sequence generation, and blinding

Allocation to treatment arms will be randomly assigned in a 2:1 ratio using permutated block randomisation. Unblinded researchers independent to the study team, utilising a simple randomisation method, will develop the randomisation sequence and assign participants to study arms. The unblinded researchers will allocate and package enema kits sequentially, and packaging will be identical to conceal treatment allocation and blinding. The study staff, investigators, trial biostatistician, nurses delivering the enemas, and participants will be blinded to group allocations.

### Data storage and management

Participants will be allocated a unique study ID number on enrolment, and their data will be de-identified and coded with this unique study ID. Blood and stool samples collected will be stored in a minus 80 °C until analysis. Faecal samples will be sent to the Queensland-based laboratory, Microba, for metagenomic analysis. DNA will be extracted and then sequenced using an Illumina NovaSeq high-throughput sequencing platform. The sequences allow the microorganisms in the samples to be classified by comparing against reference databases so that the presence and abundance of the microorganisms present can be identified and reported. Questionnaire and other study data will be collected online and stored using the secure Deakin University REDCap server.

All written and electronic data, including source documents, informed consent forms, and ethics approval forms will be retained for at least 15 years from the end of the study, as is the GCP requirement for clinical trials. All research data from consenting participants, including gut microbiota sequencing data and pre-processing and analysis pipeline information, will also be stored in a secure data repository (Deakin Research Online) for long-term preservation and/or reuse by other researchers with appropriate ethical approval/consideration from their institution (for example, meta-analyses).

### Data analysis and reporting

Reporting of findings will be in accordance with CONSORT extension for pilot and feasibility trials [59]. The study biostatistician responsible for the analysis of outcome data, as well as participants and patient assessors, will be blind to group allocation.

### Impact of and response to participant withdrawal

Participants will be withdrawn from the study if they withdraw consent or at the discretion of the researcher given adverse events or loss to follow-up. Reasons for withdrawal will be documented, and participants will be asked whether they are willing to accept any further contact to assess short- and longer-term safety.

### Management of adverse events

#### Managing risk

On enrolment, all participants will be required to provide details for their current or preferred general practitioner. In the case of a participant reporting suicidal ideation, plan, or intent, (a MADRS suicide item score of 5 or 6), the researcher will strongly encourage the participant to contact their general practitioner, and the researcher will also attempt to contact the general practitioner directly. In the case that a more serious concern for the immediate safety of the patient arises, such as severe suicidality with immediate risk, or an unreported recent suicide attempt, the researcher will contact a psychiatrist member of the team or take immediate action to ensure participant safety. This may involve—with the participant’s consent—contacting the treating general practitioner or other treating clinician, assisting the person in accessing relevant care, for instance by contacting ambulance or psychiatric emergency services.

#### Safety requirements

Study participants will be provided with contact details for the research team, and strongly encouraged to contact the research team at any point in the study to report adverse events or discuss concerns. In addition to this, there will be routine contact made every 2 weeks post-intervention until the 8-week primary endpoint, and an additional 26-week follow-up, to assess adverse events or side effects. In the circumstance of a severe or serious adverse event, participants will be encouraged to attend medical services.

If a severe or serious adverse event occurs, the re-identification protocol may need to occur, so that treating clinicians can know whether the participant received the FMT intervention or the placebo. Each participant will be issued a unique identifying number on enrolment which will be linked to the intervention that they were allocated. The unblinded research assistant will be contacted to reveal the treatment allocation of the participant, only if necessary for their medical care.

A data safety monitoring board (DSMB) will be implemented comprising of a clinician, a statistician, and a pharmacist. All members of the DSMB will be independent of the research to avoid conflicts of interest. The role of the DSMB will be to oversee the safety of the study. This will involve reviewing the study protocol, PICF and consent procedures, safety monitoring procedures, procedures relating to confidentiality and adverse events arising during the course of the study. In the event of a severe or serious adverse event, the DSMB will be promptly notified and will decide on whether to pause recruitment or halt the study.

## Ethical, regulatory, and dissemination aspects

### Consent and enrolment

Following primary expression of interest, via any of the above listed recruitment pathways, eligible participants will be directed to the study webpage for more details, where they will be able to register an expression of interest to be contacted by a member of the study team. Potentially eligible participants will be provided with a PICF. They will also be asked to nominate a time for a member of the research team to call them to discuss the study further and book in an initial study appointment. Participants will be given the option of signing the PICF with a member of the research team either in-person or via telehealth. Signed consent must be provided prior to study enrolment.

Consent will also be obtained from participant to send reminder texts prior to appointments.

Should participants have any conditions or commitments that preclude them from participating immediately, participants will be asked to nominate their preferred starting date within the trial period. Consent will be obtained to send participants reminders via email and phone.

Consent will be confirmed again at the initial screening appointment. Participants will be made aware that they can withdraw consent (primary or extended) at any point

### Plans for return of results of research to participants

At the conclusion of this study, a summary of results including aggregated results and their personal microbiome analysis report will be returned to participants via email or postal mail depending on their preference.

### Plans for dissemination and publication of project outcomes

After approval by the coordinating principal investigator, sub-investigators, and statistician, results of the study will be published in peer-reviewed scientific journals and presented at scientific conferences and to lay audiences where applicable. Results will be published after termination of the study. The order of authors will be at the discretion of the coordinating investigators. Factors that the coordinating investigator may take into consideration are the following: securing funding for the study, participation in organising the study, participation in meetings and the on-going development of the study, manuscript preparation, and general involvement in the study.

### Project closure processes

At the conclusion of the project, arrangements shall be made for retention of all data relating to the project (including raw and electronic data, and PICFs); this will be retained for at least 15 years following the closure of the database. With participant consent, biological products will be retained for up to 15 years, and contact details will be kept on record so that participants can be contacted again for participation in future research.

### Plans for sharing and/or future use of data and/or follow-up research

This project is inter-departmental and interdisciplinary. Data will be shared between the respective groups involved in its execution. There are plans for future use of the data and product developed in this project. If this project proves to be feasible, our group intends to use the same or similar enema FMT product and protocol and test its efficacy in a larger randomised controlled trial powered to detect changes in depressive symptomatology.

## Discussion

Despite the promising body of evidence and biological rationale to support the efficacy of FMT in MDD, the known relationship between intestinal dysbiosis and MDD [7, 23, 66–68], and the established efficacy of FMT in altering intestinal microbiota [69–71], there have been no published studies to date examining the impact of FMT on MDD in humans. FMT may represent a safe, fast, and effective way of treating MDD. This is potentially highly significant given the enormous psychosocial and economic burden of depression, and the limitations of existing treatment options. These factors provide compelling support for investigating the use of FMT in MDD.

As described above, substantial phase I evidence already exists for the safety of FMT across a broad range of indications [29], including immune-compromised populations [49] and in the long term [51]. This study is the first randomized, triple-blind, placebo-controlled feasibility trial assessing the feasibility, tolerability, and acceptability of FMT in the treatment of MDD in adults.

The present study has a long follow-up period (6 months), allowing for longer-term safety data and monitoring. The results of the present study will have multiple applications. Firstly, if the study is found to be feasible, they will be used to support and inform a fully powered efficacy study, which will also utilise a randomised, triple-blind, placebo-controlled design. Secondly, the study will allow us to determine whether enema-delivered FMT is acceptable to people with depression; this is important given the nature of this intervention, with the requirement to attend a clinic on four consecutive days to receive an intervention. Thirdly, assessing changes in gut microbiota and the degree to which any gut microbiota changes are concordant with the donor microbiota may yield useful information regarding dosing and frequency. Moreover, examination of possible biomarker changes that are linked to MDD, and in many cases the gut microbiota, may yield insights into mechanistic processes prompted by FMT. Fourth, although not powered to detect efficacy endpoints, some initial suggestions of potential changes in symptomatology and measures of well-being and functioning may be useful in understanding the sample size required for a scaled RCT. Finally, collecting and analysing initial cost-efficacy data will also inform the next steps in the application of this intervention in a clinical setting.

Choice of enema as a delivery route was based upon numerous factors, including feasibility, resource utilisation, safety, tolerability, patient preference, and efficacy. The most poorly tolerated and highest risk deliveries of FMT are via colonoscopy or nasogastric/nasoduodenal delivery [28]. Nasogastric/nasoduodenal delivery methods carry small but non-zero risks of perforation, aspiration, and vomiting, and are technically more complex to perform. Whilst considered the most efficacious, the colonoscopic route is also complex to perform in a research setting; this process requires a gastroenterologist, is more resource intensive, invasive, and has theoretical risks associated with use of sedation, bowel preparation and a risk of perforation, notwithstanding limitations in translation and dissemination [72]. On the other hand, whilst encapsulated FMT is considered a safe and efficacious route [73, 74], this product is not yet widely available in Australia.

Enema delivery is a widely used route of administration for FMT. It has several advantages including availability, safety, tolerability, and efficacy [72]. Additionally, FMT via enema does not require clinician-specific expertise to administer, is more affordable, and generally considered a more feasible option for delivery [72]. FMT via enema has been demonstrated to be efficacious at restoring gut microbiota following disruption by antibiotics [75]. Moreover, it has been shown to be safe and efficacious in the long term [76], and even in higher risk populations such as children [50]. Therefore, in balancing these many considerations, we have chosen FMT via enema as the route of administration for our current study.

The primary limitation of this study is the small sample size, meaning that the study will not be powered to statistically measure any of the intended secondary outcomes, and for this reason these are considered exploratory outcomes rather than secondary outcomes. However, the primary aim of the program is to explore feasibility, which the study has been designed to evaluate. If found to be feasible, a full-scale RCT is planned which would be adequately powered to evaluate efficacy as a primary outcome and will be powered to evaluate the secondary and exploratory outcomes of this feasibility study.
